# A duplex one-step recombinase aided PCR assay for the rapid and sensitive detection of the isoniazid resistance genes katG and inhA in *Mycobacterium tuberculosis*

**DOI:** 10.3389/fmicb.2025.1548965

**Published:** 2025-03-13

**Authors:** Zhiqiang Han, Xichao Ou, Ruiqing Zhang, Xiaona Lv, Yuxin Wang, Hongyi Li, Xinxin Shen, Xuejun Ma, Yanqing Tie

**Affiliations:** ^1^Hebei Medical University, Shijiazhuang, Hebei, China; ^2^Department of Clinical Laboratory, Hebei General Hospital, Shijiazhuang, Hebei, China; ^3^National Key Laboratory of Intelligent Tracking and Forecasting for Infectious Diseases, NHC Key Laboratory of Medical Virology and Viral Diseases, National Institute for Viral Disease Control and Prevention, Chinese Center for Disease Control and Prevention, Beijing, China; ^4^National Center for Tuberculosis Control and Prevention, Chinese Center for Disease Control and Prevention, Beijing, China; ^5^Hebei North University, Zhangjiakou, Hebei, China

**Keywords:** *Mycobacterium tuberculosis*, isoniazid resistance mutation, recombinant enzyme-assisted PCR (RAP), locked nucleic acid technology (LNA), highly sensitive

## Abstract

**Objectives:**

Drug resistance in tuberculosis seriously affects the eradication of tuberculosis, and isoniazid resistance is the second most commonly observed drug resistance in patients with tuberculosis. Timely and accurate detection of isoniazid resistance is critical to the treatment of tuberculosis.

**Methods:**

A duplex one-step recombinase-aided PCR (DO-RAP) assay was developed for the rapid and sensitive detection of the katG Ser315Thr and inhA-15 (C-T) mutations in *Mycobacterium tuberculosis*, which are the most common isoniazid-resistant mutations. Quantitative recombinant plasmids were used to evaluate the sensitivity of DO-RAP, and 91 *Mycobacterium tuberculosis* strains with different genotypes, as well as 5 common respiratory tract bacteria, were used to evaluate the specificity of DO-RAP. A total of 78 sputum specimens were simultaneously detected using DO-RAP, quantitative PCR (qPCR) and sanger sequencing of nested PCR products. Sanger sequencing results were used as the standard to verify the clinical performance of DO-RAP.

**Results:**

The reaction time of DO-RAP was less than 1 h. The sensitivity of DO-RAP was 2 copies/reaction, which was 10 times higher than qPCR. The sensitivity of DO-RAP for detecting heterogenous resistance was 5%. There was no cross-reactivity between the isoniazid wild-type gene, drug-resistant mutant genes, and other common respiratory tract bacteria. Compared with Sanger sequencing, the sensitivity, specificity, PPV and NPV of DO-RAP were all 100%. There were 7 specimens with gray zone or negative qPCR results but positive DO-RAP test results.

**Conclusion:**

The DO-RAP can be adopted in ordinary qPCR equipment for the rapid, highly sensitive and specific detection of the isoniazid resistance genes of *Mycobacterium tuberculosis*.

## 1 Introduction

Tuberculosis (TB), caused by *Mycobacterium tuberculosis* complex, is an infectious disease that ranks as a primary cause of morbidity and mortality globally. Yet in 2022, TB was the world’s second leading cause of death from a single infectious agent, after coronavirus disease (COVID-19) (World Health Organization, 2023).

Resistance to rifampicin and isoniazid (INH) characterizes multidrug-resistant tuberculosis (MDR-TB). Both MDR-TB and rifampicin-resistant TB (RR-TB) necessitate the use of second-line drug regimens for treatment (World Health Organization, 2023). Globally, INH resistance developed in 10.3% of new cases, 27.7% of treated cases, and 13.3% of pooled cases (Unissa et al., 2016). A number of studies have suggested that mono-resistance to INH is a precursor to multidrug-resistant TB (Stagg et al., 2017; O’Donnell, 2018). And most common mutations conferring isoniazid resistance are katG Ser315Thr and inhA -15 (C-T) (Jia et al., 2021; Finci et al., 2022).

Heterogeneous drug resistance (HR) is a key stage in the evolution of drug resistance in bacterial isolates. HR may arise from mixed infections, in which resistant and susceptible strains infect the same individual, or from a single clone that changes from susceptible to resistant strains due to genetic mutations under antibiotic stress (Andersson et al., 2019). The identification of isoniazid resistance, as well as the assessment of heterogeneous resistance through meticulous testing, is very essential.

Currently, the detection methods of *M. tuberculosis* drug resistance genes mainly relied on traditional PCR technology and gene sequencing. However, these methods suffer from either the low sensitivity or the high cost, complex operation, or time-consuming. To overcome these limitations, our laboratory reported previously a novel two-step recombinase-assisted PCR (RAP) method for rapid and highly sensitive detection of DNA or RNA (Fan et al., 2021; Fan et al., 2023) and a multiplex two-step RAP (MLP-RAP) method for the detection of *M. tuberculosis* rifampicin resistance genes, with a sensitivity of up to 5 copies/reaction and a heterogeneous resistance detection capability of 5% (Zhang et al., 2023). The principle of RAP involves a first-round amplification step (upper layer) using one pair of RAA primers followed by a second round of amplification (lower layer) using one pair of qPCR primers and a qPCR probe. This two-step RAP method requires the use of docosane as a physical partition to divide the reaction system into upper and lower layers. While the sensitivity of the test is enhanced compared to qPCR, it is somewhat more complex in terms of manual handling.

**Figure 1:**
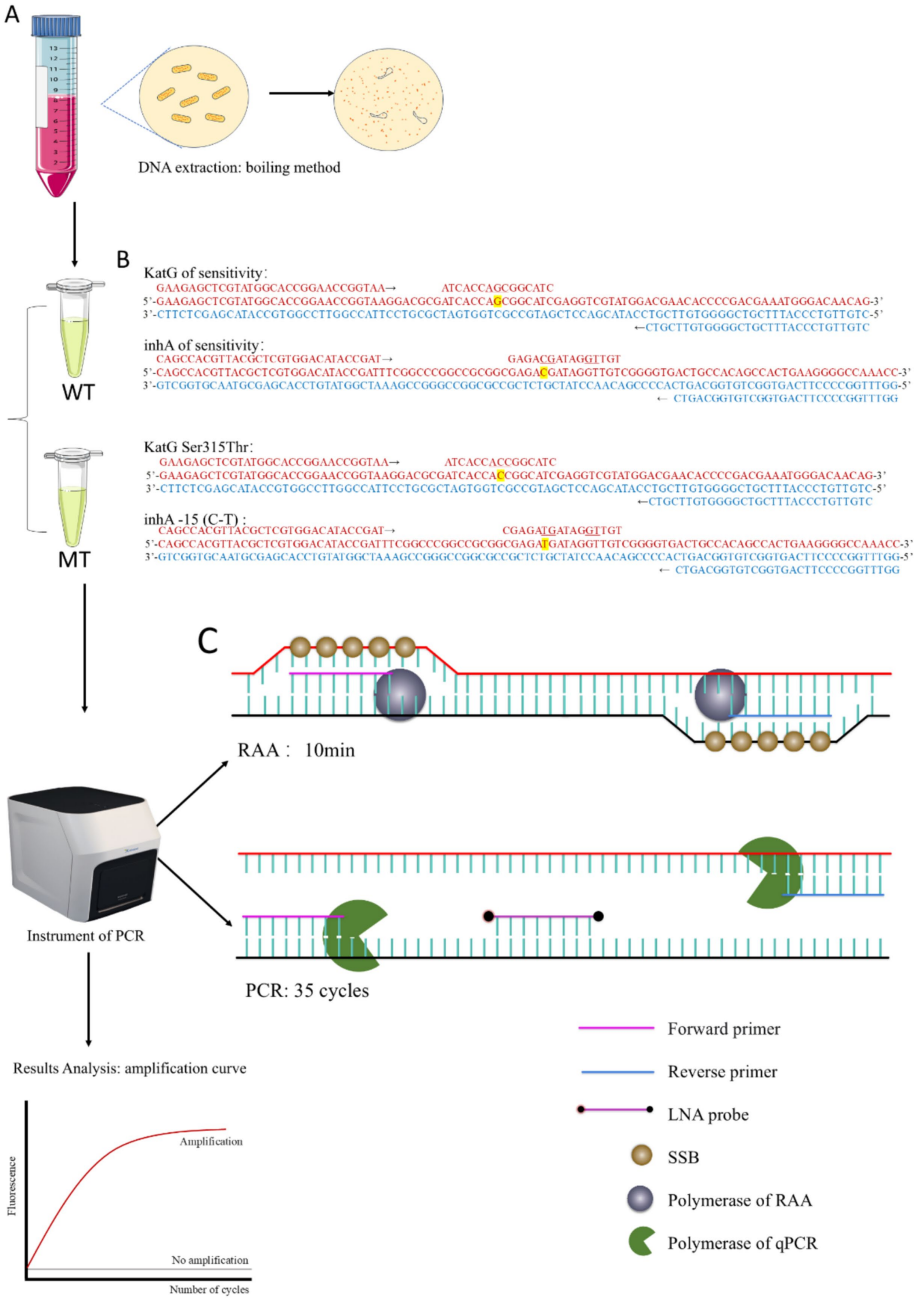
**(A)** Workflow of DO-RAP. **(B)** Design of primers and probes for WT and MT tubes. **(C)** Schematic diagram of the RAA extension stage and PCR extension stage.

In this study, we developed a one-step RAP assay by optimizing the reaction solutions, thereby resolving the compatibility issue between the RAA and PCR reaction systems. We further constructed duplex one-step RAP (DO-RAP) assays for detecting isoniazid resistance genes in *M. tuberculosis*. Briefly, we designed separately two DO-RAP assays in two tubes, one was a wild-type tube (WT) to detect the wild-type genes of katG and inhA, and the other was a mutant tube (MT) to detect the most common katG Ser315Thr and inhA -15 (C-T) isoniazid resistant mutants (Figure 1). The primary difference between the WT and MT tubes was the probe used, as all other components remained consistent across both tubes.

## 2 Materials and methods

### 2.1 Strains and specimens

**Figure 2:**
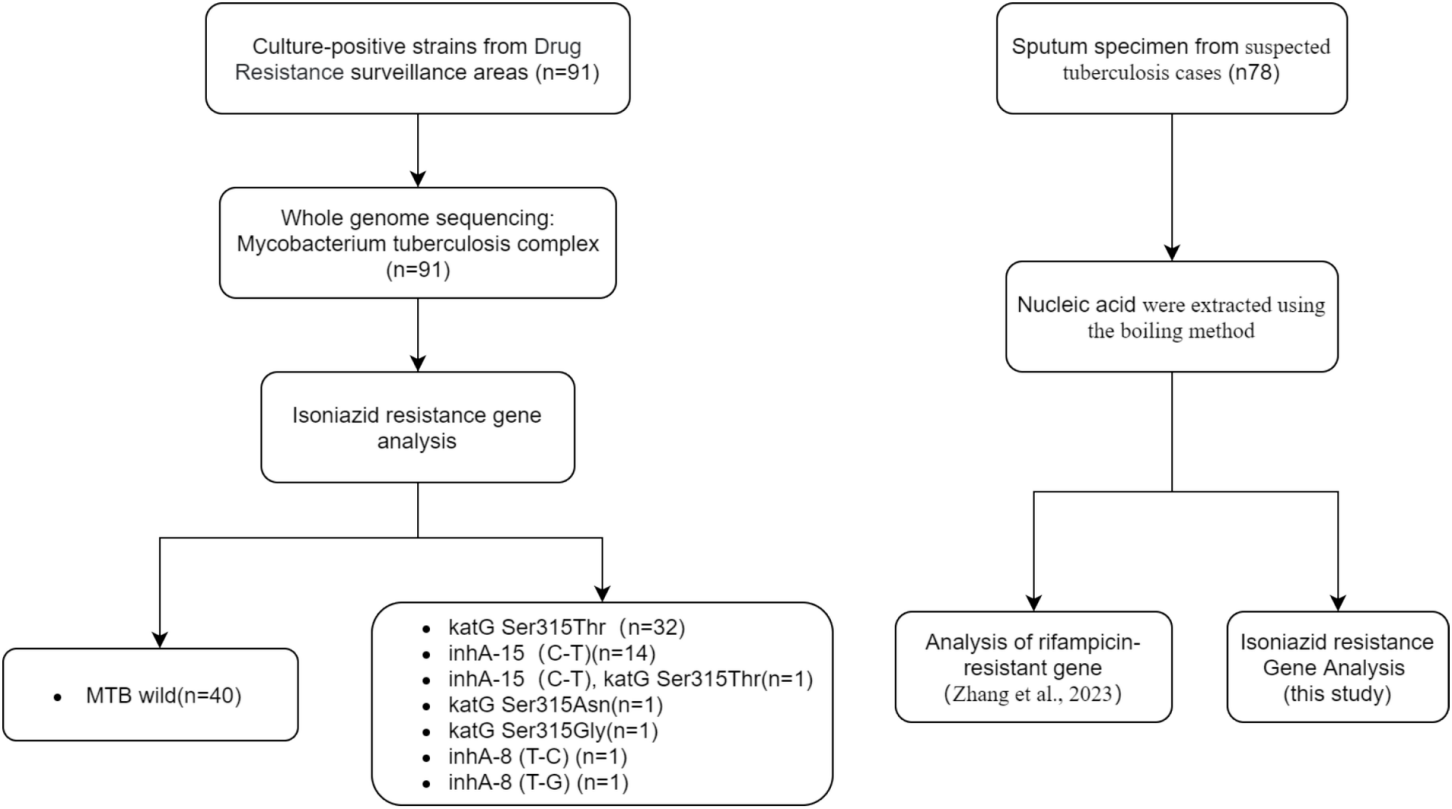
The study flow diagram for samples selection.

A total of 91clinical isolates of *M. tuberculosis*, either isoniazid-sensitive or isoniazid-resistant and 5 isolates of respiratory bacteria (including *Haemophilus influenzae*, *Streptococcus pneumoniae*, *Staphylococcus aureus*, *Klebsiella pneumoniae*, and *Pseudomonas aeruginosa*) were provided by the National Institute for Communicable Disease Control and Prevention, China CDC. These 91 isolates came from positive patients in drug resistance surveillance areas of China. The isolates were identified as *Mycobacterium tuberculosis* complex after nucleic acid extraction and whole genome sequencing. The genotypes of isoniazid resistance genes were determined as sensitive type (40 cases) and resistant type (51 cases). Additionally, a collection of 78 stocked sputum samples from suspected tuberculosis cases were provided by National Institute for Communicable Disease Control and Prevention, China CDC, and used to evaluate the clinical performance of the DO-RAP assays. The Study flow diagram for samples selection was shown in Figure 2.

### 2.2 Design of primers and probes

Sequences of katG (Gene ID: 885638) and inhA promoter (Gene ID: 886551) regions were obtained from the NCBI GenBank database. Oligo7 was used to find suitable primer and probe sequences within the gene sequences. The RAA primer candidates should have the length of 30 bp at least, and there should be no dimers between primers and between primers and probes as much as possible. The specificity of the designed primers and probes was assessed using the Primer-BLAST system at the NCBI website. Locked nucleic acid (LNA) modifications were incorporated at or near the mutation sites in the katG and inhA probes to enhance their discriminative ability for single-base mutations. The qPCR primers and nested PCR primers were selected based on the information from the literature (Peng et al., 2016). Primers and probes were synthesized by BiOligo Biotechnology (Shanghai, China) Co., Ltd. and listed in Table 1.

**Table 1:** Primer and probe information.

Gene	Primer/probe	Sequence (5′–3′)	Source
katG	katG-RAP-F	GAAGAGCTCGTATGGCACCGGAACCGGTAA	This study
	katG-RAP-R	CTGTTGTCCCATTTCGTCGGGGTGTTCGTC	This study
	katG-PCR-F	GATGGGCTTGGGCTGGAA	Peng et al. (2016)
	katG-PCR-R	AGCCGTACAGGATCTCGAGGAA	Peng et al. (2016)
	katG-PCR-OF	GGCGATGAGCGTTACAGC	Peng et al. (2016)
	katG-PCR-OR	CCAAGGTATCTCGCAACGG	Peng et al. (2016)
	katG-p-WT	FAM-ATCACCAGCGGCATC-BHQ1*	This study
	katG-p-MT	FAM-ATCACCACCGGCATC-BHQ1*	This study
inhA	inhA-RAP-F	CAGCCACGTTACGCTCGTGGACATACCGAT	This study
	inhA -RAP-R	GGTTTGGCCCCTTCAGTGGCTGTGGCAGTC	This study
	inhA -PCR-F	GGAAATCGCAGCCACGTTAC	Peng et al. (2016)
	inhA -PCR-R	TTCAGTGGCTGTGGCAGTCA	Peng et al. (2016)
	inhA -PCR-OF	CCTCGCTGCCCAGAAAGGGA	Peng et al. (2016)
	inhA -PCR-OR	ATCCCCCGGTTTCCTCCGGT	Peng et al. (2016)
	inhA -P-WT	VIC-GAGACGATAGGTTGT-BHQ1*	This study
	inhA -P-MT	VIC-CGAGATGATAGGTTGT-BHQ1*	This study

*The underline indicates the position of the LNA.

### 2.3 Recombinant plasmids preparation

The target sequences from the katG and inhA promoter regions were cloned into the pUC57 vector to create recombinant plasmids. The synthesis and construction of these plasmids were carried out by Tsingke Biotechnology Co., Ltd., Beijing, China. The engineered circular DNA molecule was measured for its amount via the Qubit^®^ dsDNA HS Assay Kit (Thermo Fisher Scientific, MA, United States) in conjunction with the Qubit2.0 fluorescence quantitative meter (Life Technologies, United States). The number of plasmid duplicates per microliter was determined by this equation: plasmid concentration (copy number/μL) = [6.02 × 10^23^ × concentration (ng/μL) × 10^−9^]/[plasmid size × 660]. Ultimately, the reference plasmids were prepared within a 1 × TE solution at a dilution series with a 10 × step, spanning from 10^0^ up to 10^5^ copies/μL. These plasmids were maintained at −80°C until their deployment.

### 2.4 DNA extraction

Totally 91 clinical isolates of *M. tuberculosis*, 5 isolates of respiratory bacteria and 78 sputum specimens were extracted using the boiling method: 500 μL of the bacterial suspension was boiled for 15 min at 100°C. After cooling, the suspension was centrifuged at 12,000 × *g* for 5 min, and 50 μL of DNA-containing supernatant was transferred to a new centrifuge tube and stored at −80°C.

### 2.5 Establishment of a one-step RAP reaction system

The incompatibility between the reaction systems of RAA and PCR arises from their differing requirements for magnesium ion concentration. RAA is compatible with magnesium ion concentrations between 7 and 14 mM, whereas the Taq enzyme utilized in standard PCR requires a narrower range, from 3 to 5 mM. Nonetheless, we hypothesize that a commercial PCR enzyme capable of functioning and tolerating an 8 mM magnesium ion concentration could potentially be utilized effectively in both the RAA and PCR reaction systems. Subsequently, we conducted a screening process to identify the enzyme that yielded the greatest increase in fluorescence within the reaction. This was achieved by testing four distinct commercial Taq DNA polymerases: RM21208 from ABclonal Biotechnology Co., Ltd. (Wuhan, China), RM29201 from ABclonal Biotechnology Co., Ltd. (Wuhan, China), PN101 from Vazyme Biotech Co., Ltd. (Nanjing, China), and ZA132TZ from GenStar Biotechnology Co., Ltd. (Beijing, China). Each enzyme was evaluated using an equivalent amount of 10^1^ copies/μL of the wild-type katG DNA plasmid as a template, within the standard reaction system that is compatible with both the RAA and PCR (RAP). The standard reaction system consisted of 10 μL of reaction buffer and RAA enzyme mixture (Lyophilized reagent, Amplification Future Biotechnology Co., Ltd., Changzhou, China), 0.25 μL of either of the above mentioned Taq DNA polymerase (5 U/L),0.75 μL of forward primer (10 μM), 0.75 μL of reverse primer (10 μM), 0.25 μL of probe (10 μM), and 9 μL of nuclease-free water, 2 μL of 100 mM magnesium ions (added to the cap of the PCR reaction tube, corresponding to 8 mM magnesium), 2 μL of extracted nucleic acid, making a total of 25 μL. The reaction program was as follows: in the first stage, the RAA reacted at 40°C for 10 min; in the second stage, the sample was pre-denatured at 95°C for 30 s followed by 35 cycles of denaturing at 95°C for 10 s and annealing at 62°C for 30 s.

### 2.6 Establishment and optimization of a DO-RAP reaction system

The use of PN101 in Methods 2.5 was determined to be the best one to achieve a single one-step RAP reaction. A DO-RAP reaction system was then established and optimized. Because of the only difference between the WT tube and MT tube is the probes, only WT tube was used to optimize the reaction conditions as follows. In the DO-RAP reaction system, primers for both the katG and inhA target genes were directly added at the same time. Considering that the increase in the number of primers may affect the reaction efficiency of the RAA, the primer concentration in the RAP reaction system and the reaction time of the RAA in the RAP program were optimized. The optimal RAA reaction time was selected by testing the plasmid at 10^1^ copies/μL with RAA reaction duration of 8 min, 10 min, and 12 min, respectively. The optimal primer concentration was determined by testing plasmids of 10^1^ with primer concentrations of 0.2, 0.3, and 0.4 μM, respectively.

### 2.7 Analytical sensitivity and specificity of the DO-RAP assay

The sensitivity of the optimized DO-RAP method and the qPCR method for detecting wild-type katG and wild-type inhA, katG Ser315Thr and inhA -15 (C-T) mutants was evaluated using corresponding wild-type or mutant recombinant plasmids with concentrations of 10^5^ to 10^0^ copies/μL. Each experiment also included a negative control with nuclease-free water. The sensitivity and reproducibility of the method were assessed by repeating the eight sensitivity tests at different times. The optimized DO-RAP reaction system and reaction procedure were described in Results 3.2. The enzyme used for qPCR was PN101 (Vazyme Biotech Co., Ltd., Nanjing, China) selected from Method 2.5. The qPCR was performed according to the instructions for the PN101. The reaction system included: 4 μL of 5 × Taq Pro Buffer, 0.2 μL of Taq Pro HS DNA Polymerase (5 U/μL), 0.4 μL of katG forward and reverse primers (10 μM), 0.2 μL of katG-p-WT or katG-p-MT 0.2 μL (10 μM), 0.4 μL of inhA forward and reverse primers (10 μM), 0.2 μL of inhA-P-WT or inhA-P-MT (10 μM), 11.8 μL of nuclease-free water, 2 μL of nucleic acid, making a total of 20 μL. The reaction procedure was as follows: 2 min at 95°C, 15 s at 95°C, and 30 s at 62°C for fluorescence collection (40 cycles).

The specificity of the optimized DO-RAP method was verified using 5 respiratory bacteria isolates (*H. influenzae*, *S. pneumoniae*, *S. aureus*, *K. pneumoniae, P. aeruginosa*) and 91 clinical isolates of *M. tuberculosis* strains that were either sensitive or resistant to isoniazid determined previously by Sanger sequencing in the National Institute for Communicable Disease Control and Prevention, China CDC.

### 2.8 Sensitivity of the DO-RAP assay for isoniazid heteroresistance

Two sets of plasmids were created, each containing a blend of wild-type and mutant DNA, which included the katG Ser315Thr and inhA -15 (C-T) mutations. These plasmid sets were prepared to have an overall concentration of 10^3^ copies/μL. The mixes were composed of varying proportions of mutant DNA, spanning from 0 to 100% with increments of 2%, 5%, 10%, 20%, 40%, 60%, and 80%. This setup was designed to evaluate the effectiveness of the MT tube in identifying infrequent mutants within a mixed template, thereby determining the limit of detection for heterogeneous resistance.

Following this, the concentration of the total DNA template was lowered to 10^2^ copies/μL. Mixtures of plasmids containing varying percentages of mutated DNA—ranging from 0 to 100%, in increments of 2%, 5%, 10%, 20%, 40%, 60%, and 80%—were once again subjected to detection using the MT tube.

### 2.9 Clinical performance of the DO-RAP assay

The practical performance of the DO-RAP assay in detecting drug resistance in *M. tuberculosis* was assessed using 78 sputum specimens. The results of the DO-RAP assay were compared with those of the real-time qPCR assay and sanger sequencing of nested PCR products. The primers used in the first round of nested PCR were katG-PCR-OF and katG-PCR-OR or inhA-PCR-OF and inhA-PCR-OR. The primers used in the second round of nested PCR were katG-PCR-F and katG-PCR-R or inhA-PCR-F and inhA-PCR-R (Table 1). The nested PCR was performed according to the instructions for the PN101. Two rounds of nested PCR assays were performed under the same operating conditions as follows: 95°C for 2 min, 45 cycles at 95°C for 15 s, 60°C for 30 s, and 72°C for 30s, each containing a negative control (water). The nested PCR products were sent to Sangon Biotech Co., Ltd. (shanghai, China) for sanger sequencing.

Statistical analyses were performed using IBM SPSS Statistics 21 (IBM Corporation, USA), and the agreement between the results of the different assays was measured using the Kappa test.

## 3 Results

### 3.1 Establishment of a one-step RAP reaction system

The highest fluorescence increment within the reaction curve was detected when using PN101 from Vazyme to identify a plasmid at a concentration of 10^1^ copies/μL. This finding suggests that PN101 is optimally adapted to the high magnesium ion concentration present in the RAP reaction system, outperforming the other three enzymes tested (Supplementary Figure 1).

### 3.2 Establishment and optimization of a DO-RAP reaction system

The RAA stage was more effective with a reaction time of 10 min compared to 8 min and 12 min (Supplementary Figure 2A). During the RAA stage, as the reaction time increased from 8 min to 12 min, the peak time of the PCR stage reaction curve progressively shifted to an earlier point. However, when the RAA reaction time was 12 min, the fluorescence increment of the reaction curve was very small, most likely because the longer RAA reaction time leads to more primer dimerisation, which interferes with the subsequent PCR reaction.

The optimal concentration of primer was 0.3 μM (Supplementary Figure 2B). As the primer concentration increased from 0.2 to 0.4 μM, both the peak time of the reaction and the fluorescence increment progressively increased. However, when the primer concentration was 0.4 μM, 10^1^ copies/μL plasmid could not be detected. The hypothesis was that this recurrence was attributed to the higher primer concentration, which led to an increase in primer dimer formation, consequently impacting the reaction’s sensitivity.

The optimized DO-RAP reaction system for WT tubes was: 10 μL of reaction buffer and RAA enzyme mixture (Lyophilized reagent), 0.25 μL of PN101 DNA Taq enzyme (5 U/L),0.75 μL of katG-RAP-F (10 μM), 0.75 μL of katG-RAP-R (10 μM), 0.25 μL of katG-p-WT (10 μM), 0.75 μL of inhA-RAP-F (10 μM), 0.75 μL of inhA-RAP-R (10 μM), 0.25 μL of inhA-p-WT (10 μM) and 7.25 μL of nuclease-free water, 2 μL of 100 mM magnesium ions (added to the cap of the PCR reaction tube, corresponding to 8 mM magnesium), 2 μL of extracted nucleic acid, making a total of 25 μL. The reaction system of the MT tube differed from that of the WT tube only by the probe, and the other components were consistent.

The optimized reaction program of DO-RAP is as follows in the first stage, the RAA reacted at 40°C for 10 min; in the second stage, the sample was pre-denatured at 95°C for 30 s; in the third stage, the sample was denatured at 95°C for 10 s and annealed at 62°C for 30 s, and the process was repeated for 35 cycles.

### 3.3 Analytical sensitivity and specificity of the DO-RAP assay

**Figure 3:**
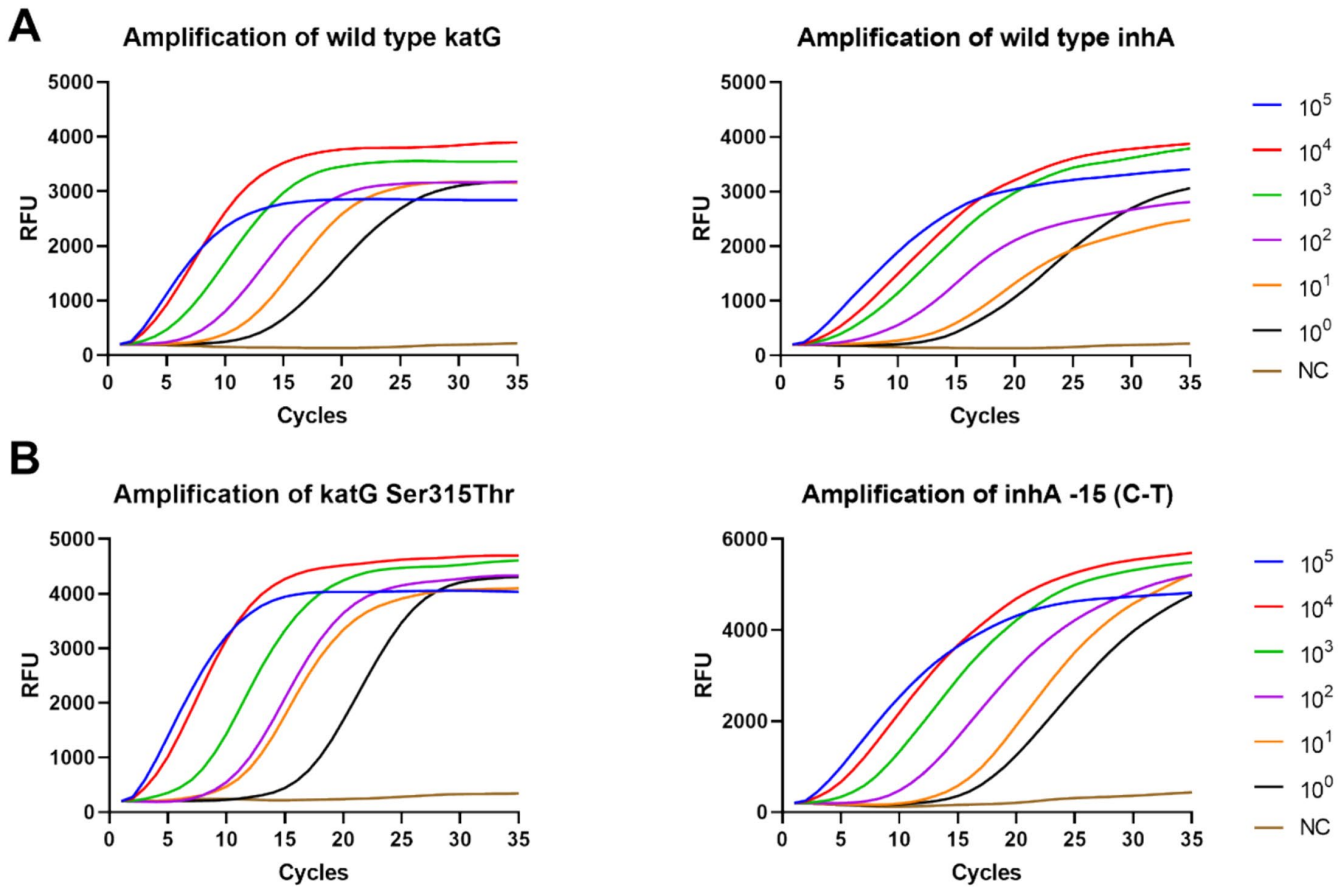
Sensitivity of the DO-RAP for detecting gradient concentration plasmids. **(A)** Amplification curve of WT tube detecting 10^5^ to 10^0^ copies/μL wild-type plasmid. **(B)** Amplification curve of MT tube detecting 10^5^ to 10^0^ copies/μL mutant plasmid.

The sensitivity of the DO-RAP and qPCR was verified using a continuously diluted recombinant plasmid. The data showed that the sensitivity of the DO-RAP for both the WT and MT tubes was 2 copies/reaction for the plasmid (Figure 3). Correspondingly, the sensitivity of the PCR was 20 copies/reaction (Supplementary Figure 3), and the sensitivity of the DO-RAP was 10 times higher than that of the qPCR.

The specificity results of DO-RAP showed that 40 strains of wild-type *M. tuberculosis* had an increase in fluorescent signal only in the WT tube. The 32 strains of katG Ser315Thr, 14 strains of inhA-15 (C-T), and one strain of both showed fluorescent signals at their corresponding locations. The results of DO-RAP were consistent with the results of sanger sequencing. The four other types of mutations (katG Ser315Asn, katG Ser315Gly), TB inhA-8 (T-C), TB inhA-8 (T-G) did not have corresponding probes designed for them, so there was only an increase in fluorescence signal at the corresponding position of the WT tube. This indicates that DO-RAP has good specificity when identifying wild-type and mutant genes. The results were all negative for the 5 common respiratory tract susceptible bacteria, indicating that there is no cross-reaction between *M. tuberculosis* and other common respiratory tract bacteria (Table 2).

**Table 2:** Specificity of the DO-RAP.

Isolated strain	Number	Result of WT tube		Result of MT tube	
		Wild type of katG	Wild type of inhA	KatG Ser315Thr	inhA −15(C-T)
MTB wild	40	Positive	Positive	Negative	Negative
MTB katG Ser315Thr	32	Negative	Positive	Positive	Negative
MTB inhA-15(C-T)	14	Positive	Negative	Negative	Positive
MTB inhA-15(C-T), katG Ser315Thr	1	Negative	Negative	Positive	Positive
MTB katG Ser315Asn	1	Negative	Positive	Negative	Negative
MTB katG Ser315Gly	1	Negative	Positive	Negative	Negative
MTB inhA-8 (T-C)	1	Positive	Negative	Negative	Negative
MTB inhA-8 (T-G)	1	Positive	Negative	Negative	Negative
*H. influenzae*	1	Negative	Negative	Negative	Negative
*S. pneumoniae*	1	Negative	Negative	Negative	Negative
*S. aureus*	1	Negative	Negative	Negative	Negative
*K. pneumoniae*	1	Negative	Negative	Negative	Negative
*P. aeruginosa*	1	Negative	Negative	Negative	Negative

### 3.4 Sensitivity of the DO-RAP assay for isoniazid heteroresistance

Different ratios of mixed plasmids were used to verify the ability of DO-RAP to detect heterogeneous resistance to isoniazid. The results showed that the MT tube was able to detect 5% of the mutant plasmids mixed with the sensitive plasmids, regardless of whether the total concentration of recombinant plasmids was 10^3^ copies/reaction or 10^2^ copies/reaction (Supplementary Figure 4).

### 3.5 Clinical performance of the DO-RAP assay

Seventy-eight sputum samples were detected by both DO-RAP and qPCR methods. qPCR results showed that 38 were sensitive, 0 were drug-resistant, 4 were gray (CT value >38) and 36 were negative. DO-RAP results showed that 45 samples were sensitive, 0 samples were resistant, and 33 samples were negative. Seven samples had qPCR results that were gray zones or negative, while the DO-RAP and Sanger sequencing results were positive. Compared with Sanger sequencing of nested PCR product assay, the sensitivity, specificity, positive predictive value (PPV), and negative predictive value (NPV), Kappa value of qPCR were 84.44%, 100%, 100%, 82.50%, 0.821, respectively. The sensitivity, specificity, PPV and NPV of DO-RAP were all 100%, and the kappa value was 1 (Table 3).

**Table 3:** Clinical performance of DO-RAP in 78 sputum samples compared with qPCR results.

		Sanger sequencing				Performance characteristics				
		Wild	Mutation	Negative	Total	Sensitivity (%)	Specificity (%)	PPV (%)	NPV (%)	Kappa
qPCR	Wild	38	0	0	38	84.44	100	100	82.5	0.821
	Mutation	0	0	0	0					
	Negative	7	0	33	40					
	Total	45	0	33	78					
DO-RAP	Wild	45	0	0	45	100	100	100	100	1.000
	Mutation	0	0	0	0					
	Negative	0	0	33	33					
	Total	45	0	33	78					

## 4 Discussion

Traditional drug susceptibility tests are based on bacterial culture to determine drug resistance, including proportion method, absolute concentration method and inhibition rate method. However, *M. tuberculosis* grows slowly, so the detection cycle is long and the operation is cumbersome, which cannot meet the needs of rapid clinical diagnosis and treatment (Shah et al., 2020). An increasing number of diagnostic methods based on rapid nucleic acid testing have sufficient sensitivity and specificity to detect genetic mutations associated with drug resistance to one or more drugs (Naidoo et al., 2023). The HAIN Line Probe Assay LPA (MTBDR) is the only rapid molecular test recommended by the WHO for detecting drug-resistant strains of *M. tuberculosis* to second-line anti-tuberculosis drugs (Maya et al., 2024), which requires advanced laboratory infrastructure and skilled operators (de Vos et al., 2018). Xpert MTB/RIF, the global standard genetic diagnostic tool for drug-resistant TB, can only detect resistance if the combination contains at least 65.6% mutant DNA (Blakemore et al., 2010). This means that it is likely to fail to provide clinical indications at an early stage of the emergence of drug resistance. The fluorescent probe PCR melting curve method (MeltPro) allows the detection of multiple mutations in a particular gene using a single probe (Liang et al., 2018). Whole genome sequencing technology can obtain the entire information on the genome, so it can identify resistance mutations to new drugs (Operario et al., 2017). However, the high cost and operation complexity of these two technologies limit their widespread application (Acharya et al., 2020). RAA is a method that can rapidly amplify nucleic acids at 37–40°C (Ma, 2022; Zhang et al., 2022). However, when the RAA detects point mutations, it is necessary to introduce 1–2 mismatched bases at the 3′ end of the primer, (Singpanomchai et al., 2021) which will affect its sensitivity to some extent. When performing multiplex detection in RAA, a pair of 30 bp primers and a 45 bp probe need to be designed for each target, which increases the difficulty of the design and multiplex detection. qPCR-based method is a fast and simple method for detecting gene mutations associated with antibiotic resistance in *M. tuberculosis*. It has the advantages of being highly automated and detecting multiple drug resistance gene mutations at the same time, but the sensitivity of qPCR needs to be further improved (He and Hu, 2024).

The DO-RAP assay established in this study combines the advantages of RAA rapidity and PCR universality, with a shorter reaction time and higher sensitivity. In a one-tube DO-RAP reaction system, the two amplification procedures of RAA and PCR can be performed sequentially without opening the lid in less than 1 h, significantly reducing the reaction time compared to fluorescence PCR methods. Both the RAA and PCR stages use the same 30-bp pair of primers, which reduces the difficulty of design multiple reaction while also reducing non-specific amplification, thereby greatly improving the lower limit of detection by 10-fold compared with that of qPCR. Compared to conventional RAA detection methods, the DO-RAP assay enables 2–3 multiplex detections in a single tube, thereby achieving reduced costs and simplified operational procedures. In comparison to the whole genome sequencing (WGS) requiring sophisticated instrumentation, the DO-RAP assay utilizes conventional laboratory equipment such as standard fluorescence PCR instruments, making it better suited for primary healthcare settings. Collectively, the DO-RAP assay provides a cost-effective solution for detecting isoniazid resistance, with the costs of $5 compared to $45 for Xpert in China. In contrast to the intricate interpretation of melting curve analyses, DO-RAP assay offers clear-cut results to the operators with minimal specialized training.

Compared to the previous two-step RAP method (Zhang et al., 2023), the DO-RAP assay does not require a docosane split layer, RAA stage and PCR stage can react sequentially in one buffer system, making the operation of DO-RAP almost the same as that of PCR, which retains the advantage of high sensitivity of the two-step RAP method and simplifies the operation at the same time. In addition, the boiling method used for nucleic acid extraction in this study does not require liquefaction operation and special equipment, which is simple and convenient and suitable for use in areas with limited resources. Furthermore, we have introduced lock nucleic acid technology to identify point mutations, with a heterogeneous drug resistance sensitivity of 5%, which is higher than all other molecular detection methods on the market, though lower than the culture-based phenotypic drug susceptibility test (DST), which can detect 1% of resistant bacteria (Folkvardsen et al., 2013). Therefore, DO-RAP can identify heterogeneous drug resistance earlier, and help clinical timely adjustment of treatment strategies.

In this study, a two-tube DO-RAP assay was designed, with one tube designated as the sensitive tube (WT) for the detection of wild-type genes katG and inhA, and the other tube designated as the drug-resistant tube (MT) for the detection of the most common mutant types of isoniazid resistance, katG Ser315Thr and inhA -15 (C-T). Through mutual verification between the two tubes, the common or rare katG and inhA mutations as well as the heterogeneous drug resistance could be possibly identified. For example, if the inhA signal is only found in the WT tube, and the katG signal does not change in fluorescence in either the WT or MT tube, it is likely that there is another mutation in katG in the 315 region, which suggests further testing is needed in the clinic.

Nonetheless, this study has some limitations. Neither isoniazid-resistant *M. tuberculosis* or the heterogeneous resistance was detected in 78 sputum samples. Therefore, we still need to continue to collect specimens to verify the performance of DO-RAP in detecting heterogeneous drug resistance in clinical samples. In addition, DO-RAP assay can detect 2–3 target genes in a tube. However, there are more than ten mutations in *M. tuberculosis* for several genes that are targeted by different anti-tuberculosis drugs (Yang et al., 2022). To address this limitation, we are attempting to combine DO-RAP assay with microfluidic chip technology, which can automatically extract nucleic acid and complete multiple RAP reactions on a single chip to improve the detection throughput and ensure the reproducibility in detecting complex clinical samples and reduce the uncertainty caused by manual operation.

The current RAP technology still has some concerns. For instance, in multiplex reactions, primers carry cross-reactivity risks that may lead to non-specific amplification. We are attempting to address this by introducing RNase H2 enzyme into the reaction system combined with ribonucleotide-embedded primers with 3′ end blocking, which can significantly reduce non-specific amplification. Additionally, the high detection sensitivity may introduce contamination risks and false-positive results. Therefore, we are working to incorporate dUTP and UDG enzyme in the reaction system to eliminate potential contamination at the initial stage of the reaction. As to the enzyme stability, much effort has been put to optimize the freeze-drying process of reaction components to reduce the batch difference and the deterioration of reagents during transportation and storage.

In conclusion, we demonstrated that the DO-RAP method is simple to operate, highly sensitive and time-saving, which can be potentially widely used in many of the current PCR application scenarios.

## Data Availability

The original contributions presented in the study are included in the article/Supplementary material, further inquiries can be directed to the corresponding authors.
