# Supplementary material for: A duplex one-step recombinase aided PCR assay for the rapid and sensitive detection of the isoniazid resistance genes katG and inhA in Mycobacterium tuberculosis
Source: Front Microbiol. 2025 Mar 13;16:1548965. doi: 10.3389/fmicb.2025.1548965 (PMC11965886; doi:10.3389/fmicb.2025.1548965)
Supplement: Supplementary file 1 [file Data_Sheet_1.docx]

Supplementary Material


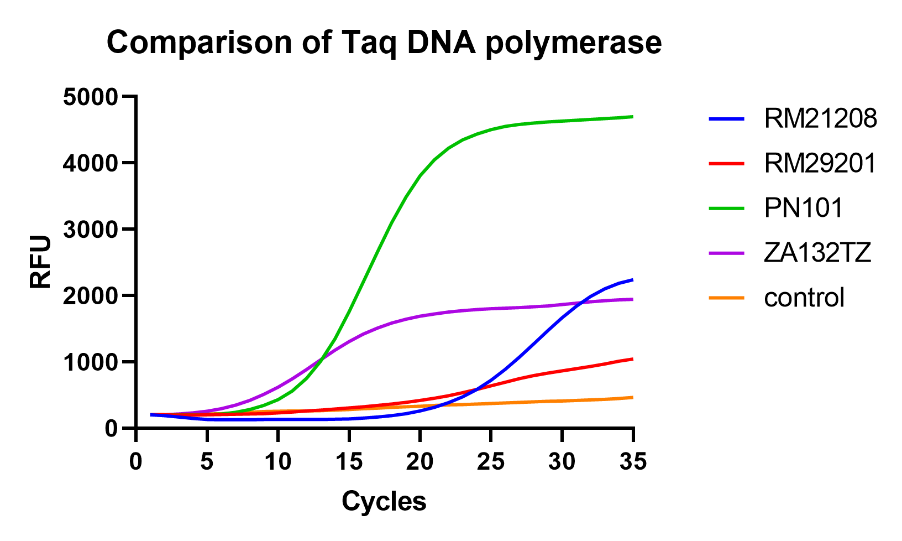


**Supplementary Figure 1.** Tolerance of different PCR DNA polymerases to the RAP reaction system.


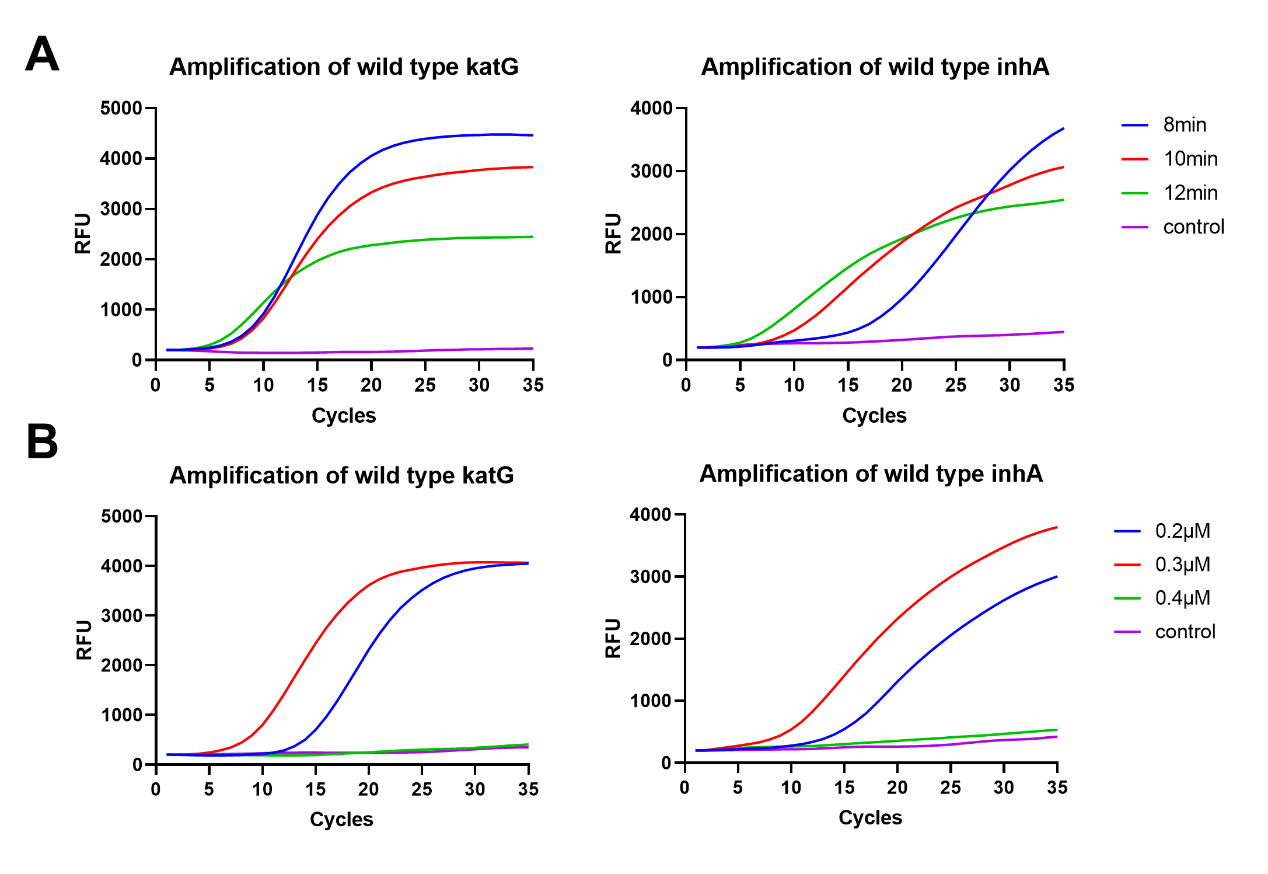


**Supplementary Figure 2.** Exploration of the reaction conditions for a DO-RAP: **(A)** DO-RAP amplification curves with different RAA incubation times. when detecting 10¹ copies /μL plasmid. **(B)** DO-RAP amplification curves with different primer concentrations when detecting 10¹ copies /μL plasmid.


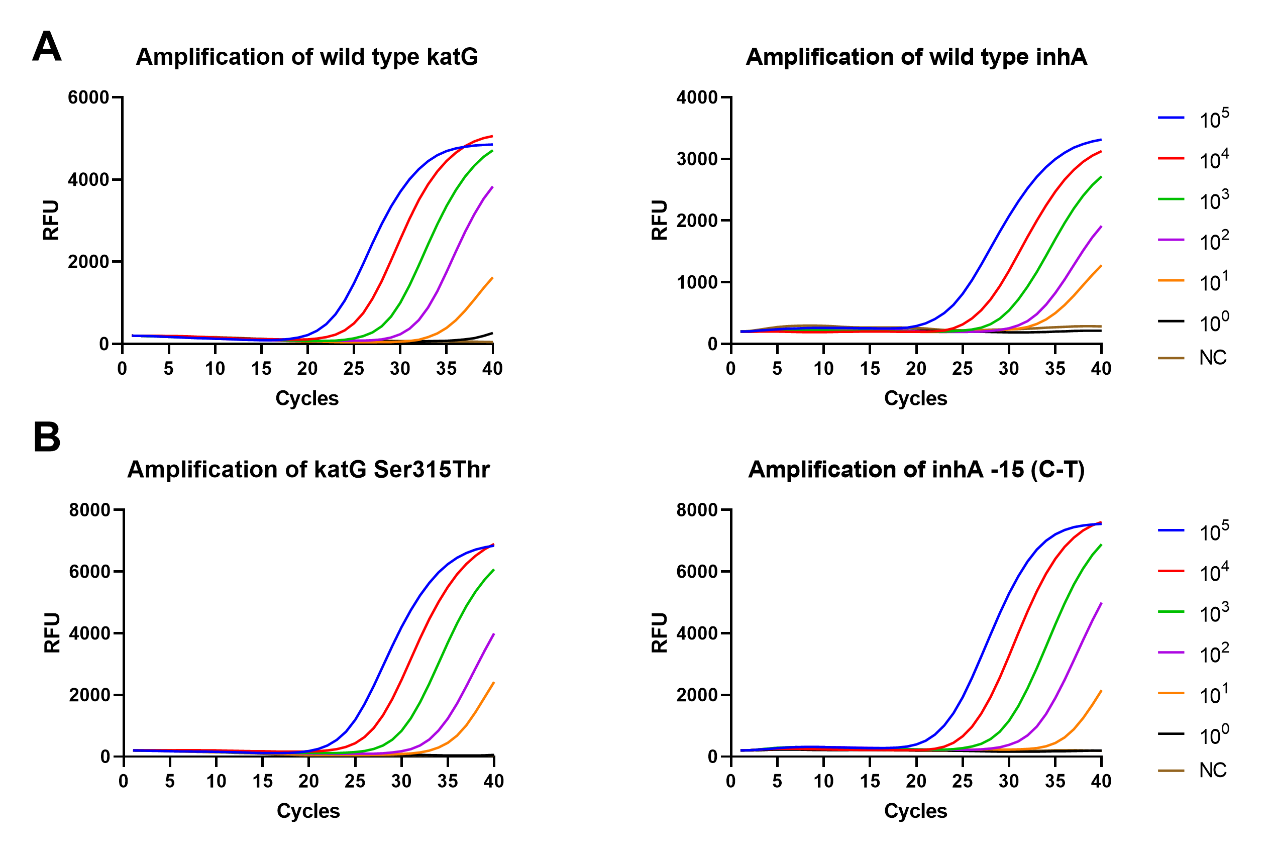
**Supplementary Figure 3.** Sensitivity of the duplex qPCR for detecting gradient concentration plasmids: **(A)** Amplification curve of duplex qPCR detecting 10⁵ to 10⁰ copies/μL wild-type plasmid. **(B)** Amplification curve of duplex qPCR detecting 10⁵ to 10⁰ copies/μL mutant plasmid.


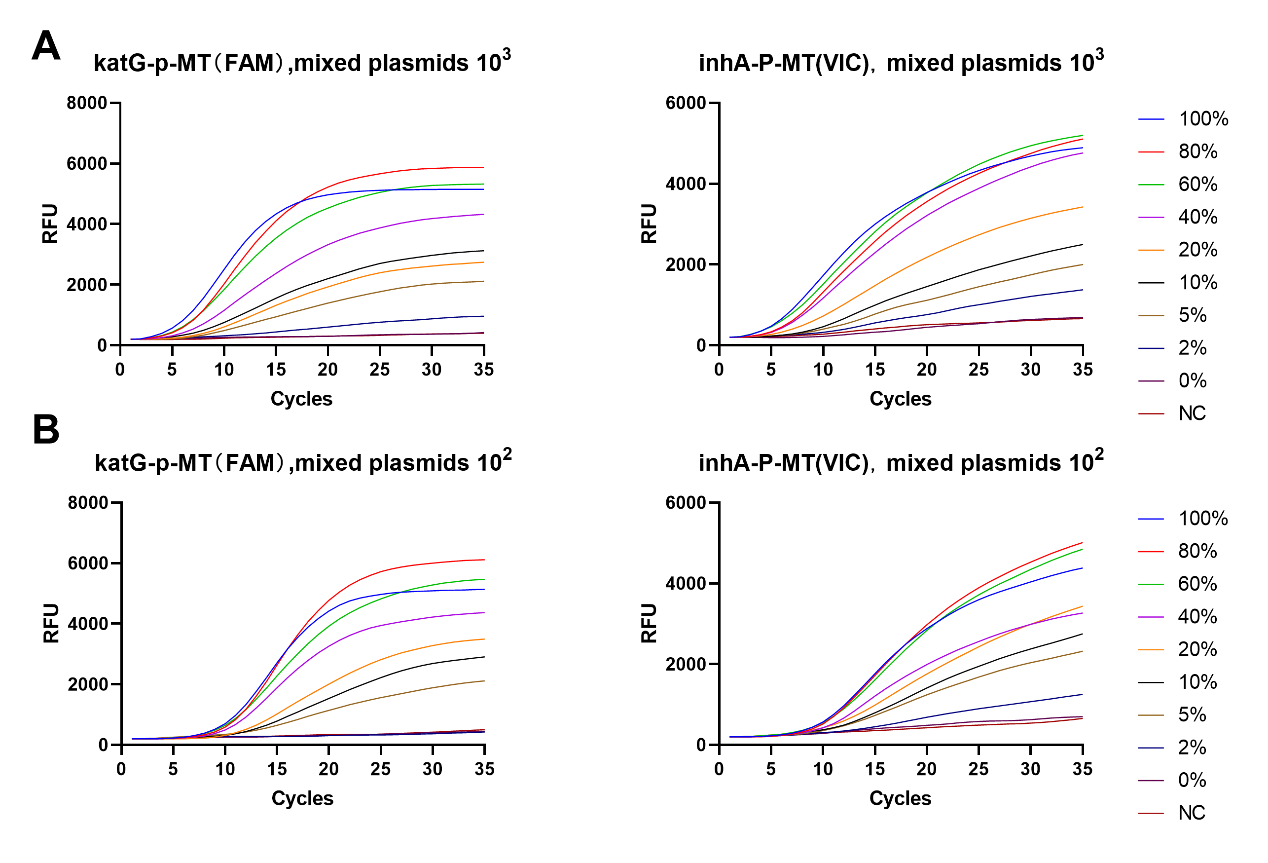
**Supplementary Figure 4.** Sensitivity of MT tubes for detection of heterogeneous resistance: **(A)** Amplification curve of MT tubes for detection of mixed plasmids at a concentration of 10³ copies /μL **(B)** Amplification curve of MT tubes for detection of mixed plasmids at a concentration of 10² copies /μL.
